# Berberine Alleviates Amyloid *β*-Induced Mitochondrial Dysfunction and Synaptic Loss

**DOI:** 10.1155/2019/7593608

**Published:** 2019-05-02

**Authors:** Chunhui Zhao, Ping Su, Cui Lv, Limin Guo, Guoqiong Cao, Chunxia Qin, Wensheng Zhang

**Affiliations:** ^1^Beijing Area Major Laboratory of Protection and Utilization of Traditional Chinese Medicine, Beijing Normal University, Beijing 100088, China; ^2^Engineering Research Center of Natural Medicine, Ministry of Education, Beijing Normal University, Beijing 100088, China; ^3^Institute of Chinese Materia Medica, China Academy of Chinese Medical Sciences, Beijing 100700, China; ^4^Laboratory of Immunology for Environment and Health, Shandong Analysis and Test Center, Qilu University of Technology (Shandong Academy of Sciences), Jinan 250014, China; ^5^Faculty of Geographical Science, Beijing Normal University, Beijing 100875, China; ^6^National & Local United Engineering Research Center for Sanqi Resources Protection and Utilization Technology, Kunming 650000, China

## Abstract

Synaptic structural and functional damage is a typical pathological feature of Alzheimer's disease (AD). Normal axonal mitochondrial function and transportation are vital to synaptic function and plasticity because they are necessary for maintaining cellular energy supply and regulating calcium and redox signalling as well as synaptic transmission and vesicle release. Amyloid-*β* (A*β*) accumulation is another pathological hallmark of AD that mediates synaptic loss and dysfunction by targeting mitochondria. Therefore, it is important to develop strategies to protect against synaptic mitochondrial damage induced by A*β*. The present study examined the beneficial effects of berberine, a natural isoquinoline alkaloid extracted from the traditional medicinal plant *Coptis chinensis*, on A*β*-induced mitochondrial and synaptic damage in primary cultured hippocampal neurons. We demonstrate that berberine alleviates axonal mitochondrial abnormalities by preserving the mitochondrial membrane potential and preventing decreases in ATP, increasing axonal mitochondrial density and length, and improving mitochondrial motility and trafficking in cultured hippocampal neurons. Although the underlying protective mechanism remains to be elucidated, the data suggest that the effects of berberine were in part related to its potent antioxidant activity. These findings highlight the neuroprotective and specifically mitoprotective effects of berberine treatment under conditions of A*β* enrichment.

## 1. Introduction

Synaptic dysfunction is an early event in the pathogenesis of Alzheimer's disease (AD), and memory and cognitive loss is more strongly correlated with synaptic dysfunction than with the development of senile plaques, neurofibrillary tangles, or gliosis [1–3]. Synapses are the basic functional unit of signal transduction in the central nervous system, forming connections and transmitting electrical and chemical signals between neurons. Accordingly, synapses are sites of high energy demand [4]. Adequate mitochondrial function is critical to meeting the high energy requirements of the synapse. Synaptic mitochondria are synthesized in neuronal soma, transported to axons and dendrites, and distributed among synapses to support synaptic function and modulate calcium homeostasis [5, 6]. Recent studies also indicate that the appropriate intracellular distribution and trafficking of mitochondria are essential for normal neuronal functions including neurotransmission, synaptic plasticity, and axonal outgrowth [7–9]. Importantly, abnormalities in mitochondrial function play an important role in AD [6].

Amyloid *β* (A*β*) is an important pathogenic peptide that is associated with AD and directly disturbs mitochondrial function [10, 11]. A*β* is transported into the mitochondria via the receptor for advanced glycation end products, the translocase of the outer mitochondrial membrane, or endoplasmic reticulum-mitochondrial crosstalk [10, 12–14]. Mitochondrial A*β* accumulation impairs mitochondrial respiration, decreases ATP production and the mitochondrial membrane potential, and increases calcium influx, cytochrome c release, and oxidative stress [15, 16]. Furthermore, the interaction of A*β* with proteins such as alcohol dehydrogenase and cyclophilin D exacerbates A*β*-induced mitochondrial and neuronal stress [17–20]. Recent studies indicate that brief exposure of cultured hippocampal neurons to relatively low concentrations of A*β* is sufficient to mediate the rapid (within 10 min) and severe impairment of mitochondrial transport in the absence of apparent cell death or significant morphological changes [21]. Taken together, these studies suggest that mitochondria are a direct site for A*β*-mediated cellular perturbation and that overt mitochondrial dysfunction occurs in an A*β*-rich environment.

Berberine is a natural isoquinoline alkaloid derived from the traditional medicinal plant *Coptis chinensis* that has numerous pharmacological properties including antimicrobial, antioxidant, anti-inflammatory, antidiarrheal, antidiabetic, antidyslipidemic, and antitumour activities [22, 23]. Recent studies have indicated that berberine treatment significantly improves memory and cognitive dysfunction in different animal models of AD [24, 25]. Due to its ability to cross the blood-brain barrier [26], berberine exerts beneficial neuroprotective effects against homocysteic acid, calyculin A, 6-hydroxydopamine, streptozotocin, and mercury-induced neurodegeneration as demonstrated through *in vivo* and *in vitro* studies [27–31]. Furthermore, berberine treatment inhibits A*β*_25–35_-induced cytotoxicity and apoptosis by suppressing the release of cytochrome C, apoptotic protein expression, and caspase activity in primary cultured hippocampal neurons [32]. Yet whether berberine has protective effects against A*β*-induced mitochondrial dysfunction and synaptic loss in neurons remains unclear. Therefore, we investigated the effects of berberine on A*β* oligomer-induced axonal mitochondrial dysfunction using an *in vitro* hippocampal neuron-cultured model.

## 2. Materials and Methods

### 2.1. Chemicals and Regents

Berberine (purity > 98%), human A*β*_1-42_, dimethyl sulfoxide (DMSO), 1,1,1,3,3,3-hexafluoro-2-propanol (HFIP), 3-(4,5-dimethylthiazol-2-yl)-2,5-diphenyltetrazolium (MTT), and penicillin/streptomycin were purchased from Sigma-Aldrich (St. Louis, MO). Neurobasal-A medium, B27, fetal bovine serum (FBS), and GlutaMax supplement for cell culture were obtained from Thermo Fisher Scientific (Waltham, MA). Berberine was reconstituted in DMSO to produce stock solution (25 mM) and then diluted with culture medium to various concentrations for cell culture experiments. The final concentration of DMSO in each sample was less than or equal to 0.004% (*v*/*v*).

### 2.2. Preparation of Oligomeric A*β*

Lyophilized human A*β*_1-42_ peptide was gently dissolved in 100% HFIP at a concentration of 1 mg/mL and quickly aliquoted into 0.1 mg stock solutions. The stock solutions were stored at room temperature and protected from light for 2–24 h before removing HFIP by evaporation, leaving a thin transparent film of peptide on the internal surface of the tube. Then, HFIP-treated peptide was dissolved in anhydrous DMSO at 5 mM, sonicated in a water bath for 10 min, and diluted to 100 *μ*M in PBS (pH 7.4). Diluted peptides were then incubated for 24 h at 4°C to obtain oligomeric A*β*_1-42_ [33].

### 2.3. Primary Hippocampal Neuron Culture and Drug Treatment

One-day-old male C57BL/6 mice (Vital River Laboratory Animal Technology, Beijing, China) were used in this study. The mice were sacrificed by decapitation, and primary neuronal cultures were prepared from the hippocampi. Briefly, hippocampi were dissected and collected in ice-cold D-Hanks solution, then treated with 0.05% (*v*/*v*) trypsin for 20 min at 37°C. FBS was added to terminate the digestion. The suspension was centrifuged at 800 × *g* for 10 min and resuspended in Neurobasal-A medium supplemented with 2% (*v*/*v*) B27. Cells were plated onto poly-D-lysine (10 *μ*g/mL) precoated 96-well plates or glass-bottom dishes with 4 chambers (cellVIEW, Greiner, Germany). The cells were cultured at 37°C under a humidified atmosphere of 5% CO_2_ until use. Half of the initial medium was removed on day 1 and replaced with fresh medium. Neurons were used for experiments after 14 days in vitro. In the cell viability assay, neurons were treated with different doses of berberine (0, 0.375, 0.75, 1.5, or 3 *μ*M) or oligomeric A*β*_1-42_ (0, 0.1, 0.2, 0.5, 1, 2, or 5 *μ*M) for 24 h before the incubation of MTT. In the mitochondrial membrane potential detection, intracellular ATP assay, ROS, and MDA measurement, neurons were preincubated in the absence or presence of berberine (0.1, 0.3, or 1 *μ*M) for 1 h before the addition of oligomeric A*β*_1-42_ (0.5 *μ*M) for 24 h to assess the protective effects of berberine on neurons treated with A*β*_1-42_ aggregates. In the axonal mitochondrial density and length measurement and axonal mitochondrial trafficking recording experiment, neurons were preincubated in the absence or presence of berberine (1 *μ*M) for 1 h before the addition of oligomeric A*β*_1-42_ (0.5 *μ*M) for 24 h.

### 2.4. Cell Viability Assay

To explore the cytotoxicity of berberine and oligomeric A*β*_1-42_ and to examine the effect of berberine on cell death induced by oligomeric A*β*_1-42_, cell viability was evaluated using the MTT colorimetric assay. Cells were incubated with MTT (0.5 mg/mL final concentration) dissolved in fresh complete medium for 4 h at 37°C, and metabolically active cells were visualized by the formation of formazan. Formazan crystals were dissolved in DMSO, and absorbance values were measured on a Multiskan MK3 microplate reader (Thermo Labsystems, Waltham, MA) using a reference wavelength of 630 nm and a test wavelength of 490 nm.

### 2.5. Mitochondrial Membrane Potential

Mitochondrial membrane potentials in cultured primary hippocampal neurons were determined using the JC-1 mitochondrial transmembrane potential detection kit (Cell Technology, Fremont, CA). Neurons cultured in glass-bottom dishes with 4 chambers were incubated with 1× JC-1 reagent for 20 min at 37°C under a humidified atmosphere of 5% CO_2_. Cells were then washed with assay buffer and examined using a laser-scanning confocal microscope (TCS-SPE, Leica, Germany). JC-1 monomers (green, excitation 485 nm, emission 535 nm) and JC-1 aggregates (red, excitation 550 nm, emission 600 nm) were measured. The ratio of red to green fluorescence was used to measure the mitochondrial membrane potential.

### 2.6. Intracellular ATP Assay

ATP levels were measured using the HS II ATP Bioluminescence Assay kit (Roche, Basel, Switzerland) in accordance with the manufacturer's specifications. Briefly, treated hippocampal neurons were rapidly washed with cold PBS, scraped into a dilution buffer, and transferred to microcentrifuge tubes. An equivalent volume of the cell lysis reagent was added to each tube, and tubes were incubated for 5 min at room temperature. After transferring the lysates to a black microtiter plate, a luciferase reagent was added to each sample by automated injection and measurement was started after a 1 s delay using a Flx800 fluorescence microplate reader (BioTek, VT). A standard curve was generated on the same plate.

### 2.7. Axonal Mitochondrial Density and Length Measurement

The operational procedure was the same as described previously [34]. Briefly, hippocampal neurons cultured in 4-well glass-bottom dishes were incubated with 100 nM MitoTracker Red (Thermo Fisher Scientific), fixed in 4% paraformaldehyde, permeabilized with 0.5% Triton X-100, and blocked with 10% goat serum. Then, neurons were incubated with anti-Tau antibody (1 : 500, Abcam, UK) and secondary antibody (Alexa Fluor 488, Abcam). The mitochondria were colored red fluorescence while axons were colored green. Images of the mitochondria with axons were collected using a confocal microscope and analysed using ImageJ (National Institutes of Health, Bethesda, MD).

### 2.8. Axonal Mitochondrial Trafficking Recording

The operational procedure was the same as described previously [34]. Briefly, neurons were incubated with MitoTracker Green (Thermo Fisher Scientific). And time-lapse images of axonal mitochondria were captured with a heated 37°C, 5% CO_2_ controlled stage for a total of 5 min. Stacks were processed using ImageJ software with a Kymograph plug-in under maximum intensity projection. A mitochondrion was considered stationary if the displacement was less than 2 *μ*m during the entire recording period. Mitochondrial movements (direction and velocity) were determined from the corresponding kymographs using ImageJ.

### 2.9. Reactive Oxygen Species (ROS) Measurement

Neurons were loaded with dichlorodihydrofluorescein diacetate (DCFH-DA) to detect ROS. After incubation with berberine or A*β*_1-42_, cultured hippocampal neurons were washed with PBS and incubated with 10 *μ*M DCFH-DA for 30 min at 37°C under a humidified atmosphere of 5% CO_2_. Images were collected using a confocal microscope and analysed using ImageJ.

### 2.10. Malondialdehyde (MDA) Measurement

The contents of MDA were measured using the malondialdehyde (MDA) assay kit (Nanjing Jiancheng, Nanjing, China) in accordance with the manufacturer's specifications. In brief, treated hippocampal neurons were rapidly washed with cold PBS, scraped into a dilution buffer, and transferred to microcentrifuge tubes. An equivalent volume of a cell lysis reagent was added to each tube, and tubes were incubated for 5 min at room temperature. The protein concentrations of lysates samples were determined by the BCA method, and then, samples were diluted with the assay buffer solution in the kit for the determination of MDA. The mixture was incubated for 40 min at 95°C, and absorbance was read optical density (OD) at 532 nm.

### 2.11. Neuronal Synaptic Density Analysis

The operational procedure of neurons was performed as described above (2.7.). Primary antibodies anti-MAP2 (1 : 100, Abcam) and anti-synaptophysin (1 : 200, Abcam) were diluted in blocking solution and added to each chamber and then incubated with either goat anti-rabbit (1 : 1000, Alexa Fluor 594, Abcam) or goat anti-mouse secondary antibodies (1 : 1000, Alexa Fluor 488, Abcam) at room temperature. Neurons were imaged using a Leica TCS-SPE confocal microscope and analysed using ImageJ.

### 2.12. Statistical Analysis

The data were analysed using SPSS version 20.0 (IBM, Armonk, NY) and are shown as the mean ± standard error of the mean of 4 independent experiments. Significant differences between values were determined using a one-way analysis of variance followed by least significant difference post hoc tests. The threshold for the statistical significance was *p* < 0.05.

## 3. Results

### 3.1. Berberine Prevents A*β*_1-42_ Cytotoxicity in Primary Cultured Hippocampal Neurons

**Figure 1:**
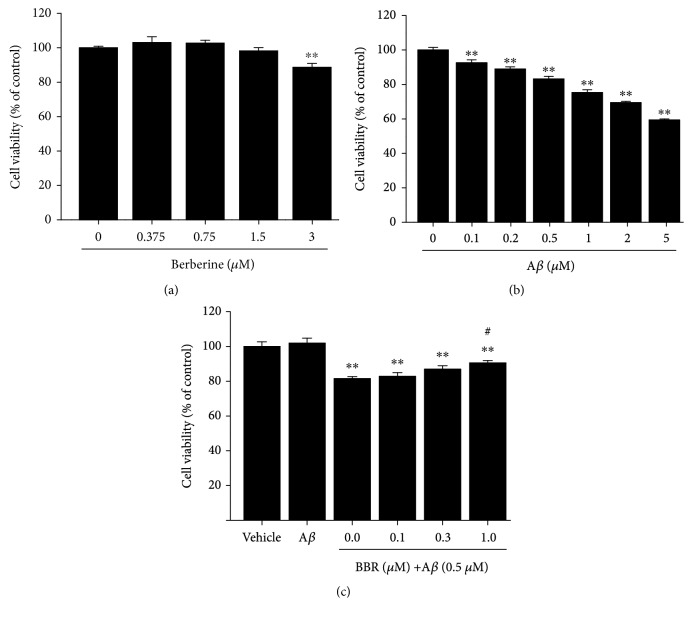
Berberine prevents oligomeric A*β*_1-42_ cytotoxicity in primary cultured hippocampal neurons. (a) Effects of berberine on cell viability. Neurons were treated with the indicated doses of berberine (0, 0.375, 0.75, 1.5, or 3 *μ*M) for 24 h. (b) Effects of oligomeric A*β*_1-42_ on cell viability. Neurons were treated with the indicated doses of A*β*_1-42_ (0, 0.1, 0.2, 0.5, 1, 2, or 5 *μ*M) for 24 h. (c) Berberine increases the viability of primary hippocampal neurons treated with A*β*_1-42_. Neurons were preincubated with berberine (0.1, 0.3, or 1 *μ*M) for 1 h and then exposed to oligomeric A*β*_1-42_ (0.5 *μ*M) for 24 h. Results were normalized to the control group (lane 1). Values are expressed as the mean ± standard error of the mean of 4 independent experiments. ^∗∗^*p* < 0.01 vs. the vehicle-treated group, ^#^*p* < 0.05 vs. the A*β*_1-42_-treated group. A*β*: cells were treated with A*β*_1-42_ at 0.5 *μ*M.

The conventional MTT reduction assay was used to examine cell viability. Cells were treated with berberine or oligomeric A*β*_1-42_ at the indicated concentrations for 24 h. Neurons treated with oligomeric A*β*_1-42_ showed a significant, dose-dependent decline in viability, while treatment with berberine at concentrations up to 1.5 *μ*M had no effect on cell viability (Figures 1(a) and 1(b)). Notably, pretreatment with berberine (0.1, 0.3, and 1 *μ*M) for 1 h before the addition of oligomeric A*β*_1-42_ (0.5 *μ*M) rescued cell viability in a concentration-dependent manner; pretreatment with 1 *μ*M berberine increased cell viability by 9.1% compared to cells incubated with oligomeric A*β*_1-42_ alone (*p* < 0.05, Figure 1(c)).

### 3.2. Berberine Attenuates the Effects of A*β*_1-42_ on Mitochondrial Membrane Potential (△ΨM) and ATP Levels in Primary Cultured Hippocampal Neurons

**Figure 2:**
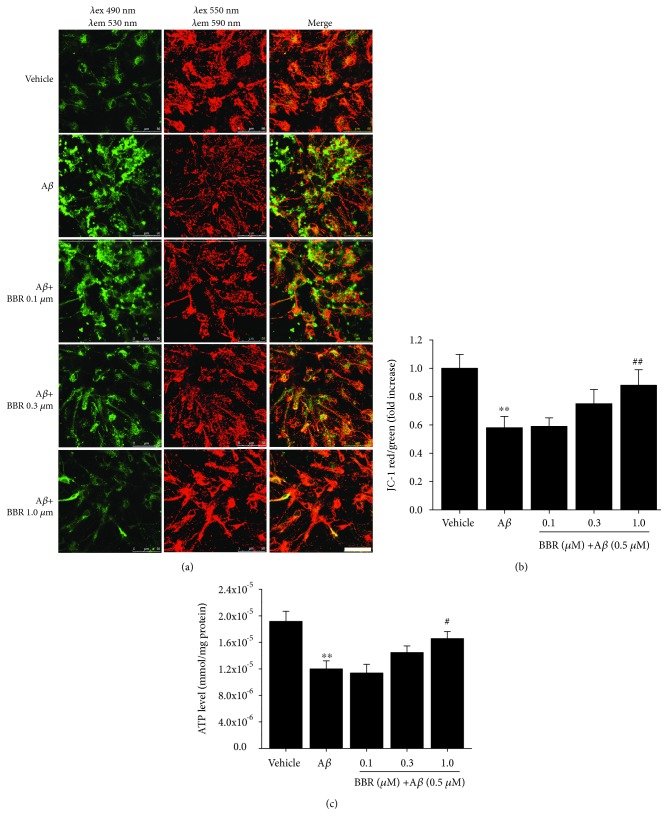
Berberine ameliorates A*β*_1-42_-induced changes in mitochondrial membrane potential and the decline of ATP levels in primary cultured hippocampal neurons. (a) Representative images of JC-1 staining of hippocampal neurons treated with vehicle, oligomeric A*β*_1-42_ (0.5 *μ*M), or oligomeric A*β*_1-42_+berberine (0.1, 0.3, or 1 *μ*M). JC-1 aggregates (red) indicate healthy mitochondria, while green fluorescence indicates cytosolic JC-1 monomers. Scale bar = 50 *μ*m. (b) The ratio of red to green fluorescence in A was quantified to measure changes in the mitochondrial membrane potential. (c) ATP levels treated with vehicle, oligomeric A*β*_1-42_ (0.5 *μ*M), or oligomeric A*β*_1-42_+berberine (0.1, 0.3, or 1 *μ*M). Values are expressed as the mean ± standard error of the mean of 4 independent experiments. ^∗∗^*p* < 0.01 vs. the vehicle-treated group, ^#^*p* < 0.05, ^##^ *p* < 0.01 vs. the A*β*_1-42_-treated group. A*β*: cells were treated with A*β*_1-42_ at 0.5 *μ*M.

To examine whether berberine could preserve mitochondrial function, we assessed △ΨM using JC-1 dye. Neurons treated with 0.5 *μ*M A*β*_1-42_ showed a significant loss of △ΨM (~42.0%, *p* < 0.01, Figures 2(a) and 2(b)). In contrast, neurons pretreated with berberine (0.1, 0.3, and 1.0 *μ*M) showed increases in △ΨM compared to neurons treated with A*β*_1-42_ alone, with a statistical significance observed for the 1 *μ*M berberine pretreatment condition (*p* < 0.01).

We next assessed the influence of berberine on neuron ATP levels after incubation with oligomeric A*β*_1-42_. As shown in Figure 2(c), ATP levels were significantly depressed in A*β*-stimulated hippocampal neurons (~37.5%, *p* < 0.01), while neurons pretreated with berberine (0.1, 0.3, 1.0 *μ*M) showed increases in ATP levels compared to neurons treated with A*β*_1-42_ alone, with a statistical significance observed for the 1 *μ*M berberine pretreatment condition (*p* < 0.05).

### 3.3. Berberine Ameliorates A*β*_1-42_-Induced Axonal Mitochondrial Fragmentation and Abnormal Trafficking in Primary Cultured Hippocampal Neurons

**Figure 3:**
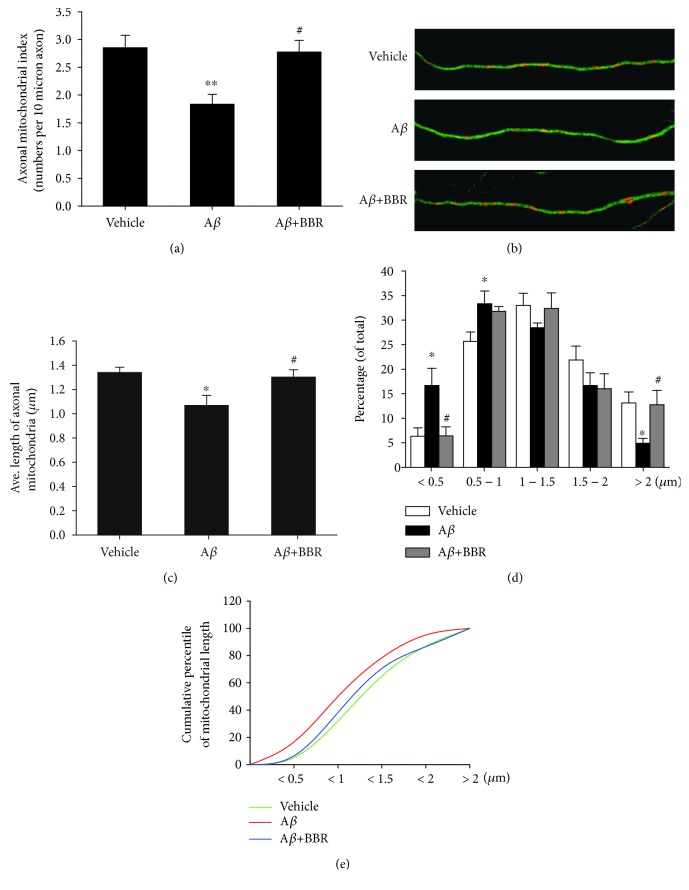
Effect of berberine on A*β*_1-42_-induced reductions in axonal mitochondrial morphology. (a) Axonal mitochondrial index (number of mitochondria per 10 *μ*m of axon) after vehicle, oligomeric A*β*_1–42_ (0.5 *μ*M), or oligomeric A*β*_1-42_+berberine (1.0 *μ*M) treatment for 24 h. (b) Representative images of mitochondrial distribution for the indicated treatment. Double immunostaining with MitoTracker Red (red) and Tau (green) are shown. Scale bar = 10 *μ*m. (c) Average lengths of axonal mitochondria after vehicle, oligomeric A*β*_1–42_ (0.5 *μ*M), or oligomeric A*β*_1-42_+berberine (1.0 *μ*M) treatment. (d) Distribution of axonal mitochondrial lengths. (e) Cumulative distribution data for the mitochondrial lengths in panel (d) ^∗^*p* < 0.05, ^∗∗^*p* < 0.01 vs. the vehicle-treated group, ^#^*p* < 0.05 vs. the A*β*-treated group. Values are expressed as the mean ± standard error of the mean of 4 independent experiments; 8 axons per group.

To further analyse the effects of berberine on A*β*-induced mitotoxicity, we next examined mitochondrial density, length, distribution, and mobility in axons. First, we counted the mitochondria (particles positive for MitoTracker Red) in axonal processes of identical lengths. Neurons incubated with A*β*_1-42_ showed a significant decrease (~35.8%) in axonal mitochondrial density compared to vehicle-treated neurons (1.833 ± 0.173 vs. 2.851 ± 0.227 per 10 *μ*m, *p* < 0.01, Figure 3(a)). In contrast, cells pretreated with 1.0 *μ*M berberine showed the rescue of axonal mitochondrial density (~51.5%) compared to neurons treated with A*β*_1-42_ alone (2.777 ± 0.209 vs. 1.833 ± 0.173 per 10 *μ*m, *p* < 0.05).

**Figure 4:**
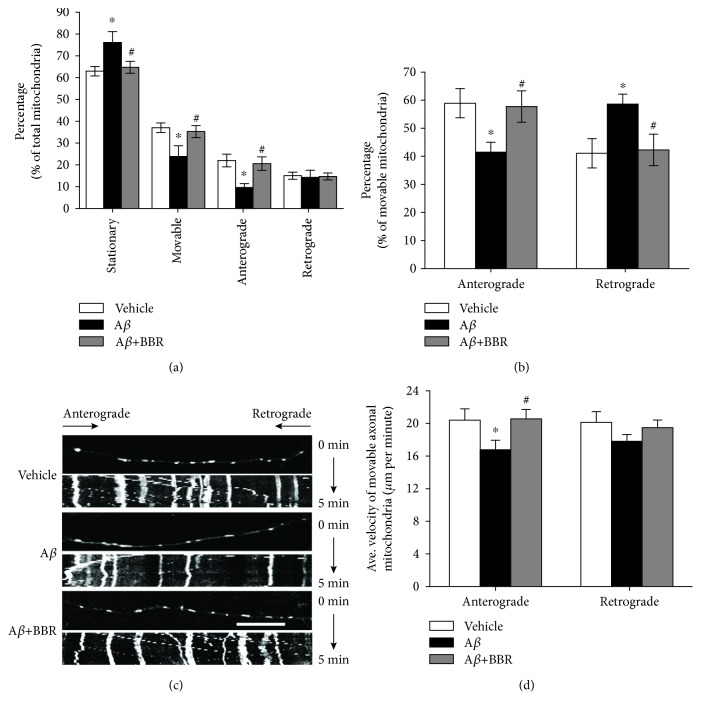
Berberine ameliorates A*β*_1-42_-induced abnormalities in mitochondrial trafficking in primary cultured hippocampal neurons. (a) Percentages of stationary, moving, moving anterograde, and moving retrograde mitochondria were compared with those of total mitochondria, respectively. (b) Percentages of anterograde and retrograde mitochondria were compared with the total amount of movable mitochondria, respectively. (c) Representative kymograph images of vehicle-, oligomeric A*β*_1–42_- (0.5 *μ*M), or oligomeric A*β*_1-42_+berberine- (1.0 *μ*M) treated axonal mitochondrial movement. Scale bar = 10 *μ*m. (d) Average transport velocity of anterograde and retrograde mitochondrial was calculated. ^∗^*p* < 0.05 vs. the vehicle-treated group, ^#^*p* < 0.05 vs. the A*β*-treated group. Values are expressed as the mean ± standard error of the mean of 4 independent experiments; 6 axons per group.

**Figure 5:**
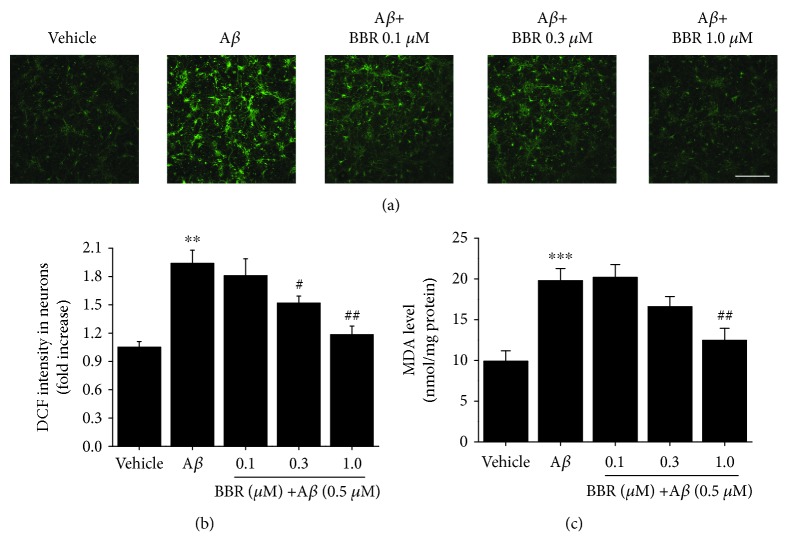
Berberine attenuates the A*β*_1-42_-induced reactive oxygen species generation and malondialdehyde elevation in primary cultured hippocampal neurons. (a) Representative images of DCF staining in hippocampal neurons for the indicated treatment. Scale bar = 250 *μ*m. (b) Quantification of DCF intensities in (a). (c) MDA were significantly elevated in A*β*_1-42_-treated neurons compared to vehicle-treated neurons and partially restored in berberine-treated neurons in a dose-dependent manner. Values are expressed as the mean ± standard error of the mean of 4 independent experiments. ^∗∗^*p* < 0.01 and ^∗∗∗^*p* < 0.001 vs. vehicle-treated group, ^#^*p* < 0.05 and ^##^*p* < 0.01 vs. the A*β*-treated group. A*β*: cells were treated with A*β*_1-42_ at 0.5 *μ*M.

Next, we evaluated changes in mitochondrial length. The average length of axonal mitochondria in A*β*_1-42_-treated neurons was significantly lower than that in the vehicle or berberine-treated neurons (1.069 ± 0.083 *μ*m in A*β*_1-42_-treated neurons vs. 1.340 ± 0.044 *μ*m in vehicle-treated neurons or 1.301 ± 0.062 *μ*m in berberine and A*β*_1-42_-treated neurons, *p* < 0.05, Figure 3(c)). Compared to vehicle-treated or berberine pretreated neurons, neurons exposed to A*β* showed a significantly higher percentage of axonal mitochondria less than 0.5 *μ*m in length and a lower percentage of mitochondria more than 2.0 *μ*m in length (Figure 3(d)). Cumulative data displayed a leftward shift in the mitochondrial length for A*β*_1-42_-treated neurons that was rescued by berberine pretreatment (Figure 3(e)). An examination of mitochondrial trafficking within hippocampal axonal processes revealed that A*β*_1-42_ treatment significantly increased the proportion of stationary mitochondria (~21%) compared to vehicle-treated neurons (76.171 ± 4.941% vs. 62.963 ± 2.169%, *p* < 0.05, Figure 4(a)). Berberine treatment partially recovered mitochondrial mobility as indicated by the proportion of stationary mitochondria (64.739 ± 2.747%, *p* < 0.05, Figure 5(a)). Conversely, the percentage of moving mitochondria, including movement in both directions (anterograde and retrograde) in A*β*_1-42_-treated neurons, was dramatically decreased compared to that of vehicle-treated and berberine-pretreated neurons (23.829 ± 4.941% in A*β*_1-42_-treated neurons vs. 37.037 ± 2.169% in vehicle-treated neurons or 35.261 ± 2.747% in berberine and A*β*_1-42_-treated neurons, *p* < 0.05, Figures 4(a) and 4(c)). Interestingly, the percentage of anterograde mitochondrial movement was slightly lower in A*β*_1-42_-treated neurons than in neurons treated with vehicle or pretreated with berberine (41.428 ± 3.577% in A*β*_1-42_-treated neurons vs. 58.930 ± 5.217% in vehicle-treated neurons or 57.729 ± 5.604% in berberine and A*β*_1-42_-treated neurons, *p* < 0.05, Figure 4(b)), whereas the percentage of retrograde mitochondrial movement was increased in A*β*_1-42_-treated neurons than in neurons treated with vehicle or pretreated with berberine (58.572 ± 3.577% in A*β*_1-42_-treated neurons vs. 41.070 ± 5.217% in vehicle-treated neurons or 42.271 ± 5.604% in berberine and A*β*_1-42_-treated neurons, *p* < 0.05, Figure 4(b)).

Finally, we measured the velocity of mitochondrial movement in axons. Consistent with our mitochondrial mobility findings, A*β*_1-42_ treatment slowed anterograde mitochondrial movement more than retrograde movement (Figure 4(d)). A*β*_1-42_ treatment decreased the anterograde velocity of axonal mitochondria by ~17.8% (16.758 ± 1.176 *μ*m/min in A*β*_1-42_-treated neurons vs. 20.396 ± 1.388 *μ*m/min in vehicle-treated neurons, *p* < 0.05, Figure 4(d)), while berberine pretreatment rescued anterograde mitochondrial velocity (16.758 ± 1.176 *μ*m/min in A*β*_1-42_-treated neurons vs. 20.561 ± 1.153 *μ*m/min in berberine and A*β*_1-42_-treated neurons, *p* < 0.05). In contrast, retrograde movement velocity was not significantly different among the 3 groups (Figure 4(d)).

### 3.4. Berberine Attenuates A*β*_1-42_-Induced ROS Generation and MDA Elevation in Primary Cultured Hippocampal Neurons

To examine the effect of berberine on A*β*_1-42_-induced oxidative stress, we measured intracellular ROS levels by measuring the fluorescence intensity of DCF in primary cultured hippocampal neurons. A*β*_1-42_ treatment significantly increased ROS levels compared to vehicle treatment (~85.0%, *p* < 0.01, Figures 5(a) and 5(b)). In contrast, pretreatment with berberine (0.1, 0.3, 1.0 *μ*M) dose-dependently decreased A*β*_1-42_-induced ROS generation. Accordingly, the MDA levels of berberine treated-neurons showed the same trend as that of intracellular ROS levels (Figure 5(c)).

### 3.5. Berberine Protects against A*β*_1–42_-Mediated Synaptic Loss

**Figure 6:**
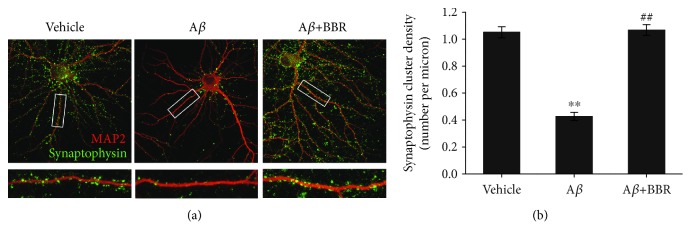
Berberine protects against A*β*_1–42_-induced synaptic loss in primary cultured hippocampal neurons. Neurons treatment with vehicle, oligomeric A*β*_1–42_ (0.5 *μ*M), or oligomeric A*β*_1-42_+berberine (1.0 *μ*M) were immunostained for MAP2 and synaptophysin. (a) Representative images for neurons and synapses showed colocalization of synaptophysin and MAP2. Scale bar = 10 *μ*m. (b) Quantification of synaptophysin immunoreactivity. ^∗∗^*p* < 0.01 vs. the vehicle-treated group, ^##^*p* < 0.01 vs. the A*β*-treated group. Values are expressed as the mean ± standard error of the mean of 4 independent experiments; 8 neurons per group.

To evaluate the protective effects of berberine on oligomeric A*β*_1–42_-induced synaptic loss as a typical pathological feature observed in postmortem AD brains, we measured synaptic density by counting synaptophysin-positive clusters (green) on dendrites. Synaptophysin-positive clusters were depleted in A*β*-treated neurons compared to vehicle- or berberine pre-treated neurons (Figure 6(a)). Synaptic density in A*β*_1–42_-treated neurons was significantly decreased by 59.4% compared to vehicle (0.427 ± 0.030 per micron vs. 1.051 ± 0.041 per micron, *p* < 0.01, Figure 6(b)), whereas the berberine treatment (1 *μ*M) reduced the negative effect of A*β*_1–42_ on synaptic density (1.067 ± 0.040 per micron vs. 0.427 ± 0.030 per micron, *p* < 0.01).

## 4. Discussion

Synapses are the basic structural foundation of signal transduction in the central nervous system, and synaptic plasticity in the brain is thought to be the cellular substrate of learning and memory. Severe synaptic structural and functional damage, which is a typical pathological features of AD, causes memory and cognitive dysfunction [34]. Mitochondria support synaptic function by maintaining the cellular energy supply, regulating calcium and redox signalling, and regulating synaptic transmission and vesicle release. Accordingly, multiple lines of evidence indicate that synaptic function and plasticity depend on the mitochondria [35]. A*β*_1-42_ is a neurotoxic peptide that induces synaptic loss by disrupting the mitochondrial membrane potential, decreasing ATP generation, enhancing intracellular ROS production, and activating mitophagy [15, 34, 36, 37]. Berberine is a natural isoquinoline alkaloid that has an array of neuroprotective properties; yet a majority of researches has focused on its beneficial effects on neurodegenerative diseases based on its antioxidant activity [23]. In the present study, we add to a previous literature by characterising berberine as a mitoprotective agent that prevents synaptic loss associated with A*β* toxicity.

The △ΨM is an important indicator of cellular health. △ΨM is generated by the proton gradient (complexes I, III, and IV) and serves as an essential component of energy storage during oxidative phosphorylation. △ΨM forms the transmembrane potential that is used by ATP synthase to generate ATP. Normally, cellular △ΨM and ATP levels are relatively stable and only undergo transient changes due to physiological activity. Yet chronic disruptions of △ΨM and ATP production compromise cell viability, leading to various pathological consequences [38]. In this study, we report for the first time the ability of berberine to ameliorate the A*β*_1-42_-induced impairment of △ΨM and ATP generation in primary cultured hippocampal neurons in a dose-dependent manner.

Because mitochondria are synthesized perinuclearly, they must be trafficked from the soma to distal synapses via mitochondrial transport and constantly reconfigure to meet synaptic needs. Moreover, mitochondrial morphology is also dynamic and can be regulated through fusion and fission. It was reported that an elongated morphology conferred bioenergetic advantages for ATP generation and dispersal [39]. Because of the technical limitations on observing mitochondrial trafficking *in vivo*, we performed studies in a primary cultured hippocampal neuronal model as reported previously [6]. We selected axonal processes for the quantitative analysis of mitochondrial length, density, distribution, and mobility because of their known morphological and dynamic characteristics [6, 34, 40]. Berberine pretreatment rescued A*β*-induced axonal mitochondrial fragmentation and abnormal trafficking, indicating that treatment supported energy homeostasis and prevented A*β*-induced mitochondrial morphological as well as functional impairment.

The exact mechanism underlying the protective effects of berberine is unclear. Various studies have established the antioxidant activity of berberine in disorders such as diabetes, high cholesterol, and CNS disorders. Berberine induces antioxidant defences by quenching superoxide anion and nitric oxide, increasing levels of non-enzymatic antioxidants and the activities of antioxidant enzymes, and upregulating Nrf2 (defends against ROS damage), GFAP, GLP-1, and other protective molecules [22, 23, 41–44]. Therefore, we examined ROS levels in primary cultured hippocampal neurons, as mitochondrial dysfunction is an important source of oxidative stress. We found that A*β*_1-42_-induced oxidative stress was decreased by berberine pretreatment, consistent with previously published data. Furthermore, berberine also has a protective effect against synaptic loss instigated by oligomer A*β*_1-42_.

This study supported the opinion that berberine could be a potential therapeutic agent for the treatment of AD, based on its effect of alleviating A*β*-induced axonal mitochondrial abnormalities and synaptic loss. However, there remain some limitations of this study requiring further investigation. One limitation is that the definite mechanism underlying protection of berberine against A*β*-induced axonal mitochondrial abnormalities remains unclear and needs to be elucidated. The mitoprotective effects of berberine may be related to its antioxidant activity which has been proven in present study, whereas antioxidants may not be the only effect and berberine may still have other potential protective mechanisms. Another limitation of this study is that protective effects of berberine against neuronal A*β* toxicity were examined only *in vitro*. We need to further verify the experimental results *in vivo*, in order to make the neuronal protective effect more credible.

## 5. Conclusions

We demonstrate that berberine protected against A*β*-induced axonal mitochondrial abnormalities in primary cultured hippocampal neurons by preserving the mitochondrial membrane potential and ATP generation, increasing axonal mitochondrial density and length, and improving mitochondrial motility and trafficking, ultimately preventing synaptic loss. Although the underlying mechanism of these effects is unclear, the data suggest that the protective effects of berberine may be related to its antioxidant activity. Future research should investigate berberine as a potential therapeutic agent in the context of AD.

## Data Availability

All data included in this study are available upon request by contact with the corresponding author.

## Abbreviations

A*β*: Amyloid beta
AD: Alzheimer's disease
DMSO: Dimethyl sulfoxide
HFIP: 1,1,1,3,3,3-Hexafluoro-2-propanol
MTT: 3-(4,5-Dimethylthiazol-2-yl)-2,5-diphenyltetrazolium
FBS: Fetal bovine serum
DIV: Days in vitro
ROS: Reactive oxygen species
DCFH-DA: 2′,7′-Dichlorodihydrofluorescein diacetate
△ΨM: Mitochondrial membrane potential.
